# Classification of breast tissue in mammograms using efficient coding

**DOI:** 10.1186/1475-925X-10-55

**Published:** 2011-06-24

**Authors:** Daniel D Costa, Lúcio F Campos, Allan K Barros

**Affiliations:** 1 Laboratory of Biological Information Processing, Department of Electrical Engineering, Federal University of Maranhão - UFMA, São Luís, MA, Brazil; 2 Institute of Engineering and Computer Science, Department of Computer Engineering, State University of Maranhão - UEMA, São Luís, MA, Brazil

## Abstract

**Background:**

Female breast cancer is the major cause of death by cancer in western countries. Efforts in Computer Vision have been made in order to improve the diagnostic accuracy by radiologists. Some methods of lesion diagnosis in mammogram images were developed based in the technique of principal component analysis which has been used in efficient coding of signals and 2D Gabor wavelets used for computer vision applications and modeling biological vision.

**Methods:**

In this work, we present a methodology that uses efficient coding along with linear discriminant analysis to distinguish between mass and non-mass from 5090 region of interest from mammograms.

**Results:**

The results show that the best rates of success reached with Gabor wavelets and principal component analysis were 85.28% and 87.28%, respectively. In comparison, the model of efficient coding presented here reached up to 90.07%.

**Conclusions:**

Altogether, the results presented demonstrate that independent component analysis performed successfully the efficient coding in order to discriminate mass from non-mass tissues. In addition, we have observed that LDA with ICA bases showed high predictive performance for some datasets and thus provide significant support for a more detailed clinical investigation.

## Introduction

Breast cancer is the major cause of death by cancer in the female population [1]. Early detection of breast cancer by mammography may lead to a greater range of treatment options, including less-aggressive surgery and adjuvant therapy [2]. Therefore, a great effort has been made to improve those techniques. Among them, the most used is the mammogram, which is simple, low cost and non-invasive. The sensitivity of mammography ranges from 46% to 88% and depends on factors such as size and location of the lesion, breast tissue density, quality of technical resources and the radiologist's ability of interpretation. The specificity varies between 82% and 99% and it also dependent on the quality of the examination [3]. The low sensitivity means that there is a considerable number of positive cases undetected, preventing early diagnosis and effective treatment.

To decrease the high index of mammogram error, during the last decade the scientific community has come to use image processing and computer-aided diagnosis (CAD) techniques to produce digital mammographies. CAD systems can aid radiologists by providing a second opinion and may be used in the first stage of examination. For this to occur, it is important to develop techniques to detect and recognize suspicious lesions and also to analyze and discriminate them. Regarding this there are some mammogram images mass classification methods in the literature. A mass might be either a benign or malignant tumor, whereas non-masses are exclusively normal tissues. Zhang et al [4] have proposed a neural-genetic algorithm for feature selection in conjunction with neural network based classifier. This methodology reached 87.2% of correct classification for mass cases with different feature subsets.

Wei [5] investigated the feasibility of using multiresolution texture analysis for differentiation of masses from normal breast tissue on mammograms and use texture features based on the wavelet coefficients and variable distances. They reached 89% and 86% of accuracy for the training and test groups, respectively. Jr et al [6] applied semivariogram function to the characterization of breast tissue as malignant or benign in mammographic images with sensitivity in 92.8%, specificity of 83.3% and accuracy above 88.0%. Land et al [7] explored the use of different Support Vector Machines (SVM) kernels and combinations of kernels, to ascertain the diagnostic accuracy of a screen film mammogram data set and improved about 4% the average of sensitivity and 18% the average of specificity, reaching 100% of sensitivity and 98% of specificity. Campos et al [8,9] used independent component analysis (ICA) and neural network multilayer perceptron to classify mammograms in 3 classes: normal, benign and malignant, obtaining a rate of 97.3% success. Braz et al [10] classified the regions of interest of screening mammogram in mass and non-mass using spatial statistics, and reached accuracy up to 98.36%.

The purpose of this work is to classify a specific region of interest (ROI) as mass or non-mass and compare different methods of *efficient coding*. This concept has successfully explained the properties of receptive fields in primary visual cortex by deriving efficient codes from the statistics of natural images [11-14]. Today this process can be modeled with ICA, which works with statistics of high order [15].

We organize this work as follows: in section II, we describe the used database, the feature extraction process with efficient coding and the classifier linear discriminant analysis. In section III we show the results obtained using the proposed methodology. Finally, section IV presents some discussions and conclusions.

## Materials and methods

**Figure 1:**
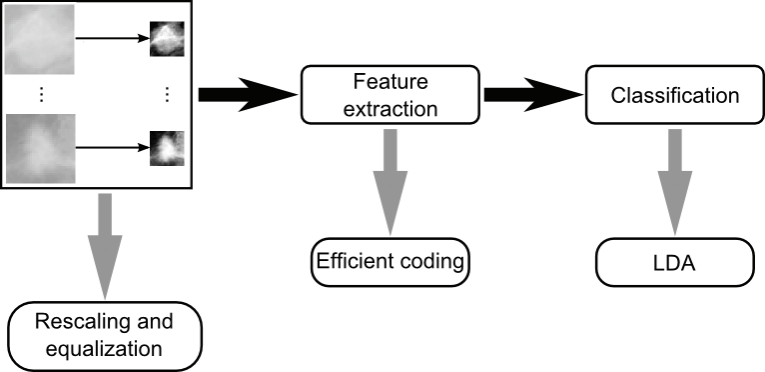
**Methodology**. Proposed Methodology based in three steps: Image acquisition, feature extraction and classification.

For a better explanation of methodology, we divide the method in three steps as described in Figure 1. The first is the image acquisition, which is made by obtaining mammograms and selecting regions that correspond to mass and non-mass. After this, a histogram equalization in each region extracted is performed to emphasize characteristics not shown in previous images.

Next we used feature extraction techniques, principal components analysis (PCA), Gabor wavelet, and the efficient coding model based in independent component analysis (ICA). In the last step, we used linear discriminant analysis (LDA) to classify these tissues as mass or non-mass. Let us describe in details each step.

### Image Acquisition

For the development and evaluation of the proposed methodology, we used a publicly available database of digitized screen- film mammograms: the Digital Database for Screening Mammography DDSM [16].

The DDSM database contains 2620 cases with two default views (medio-lateral oblique and cranio caudal) of both breasts, acquired from Massachusetts General Hospital, Wake Forest University School of Medicine, Sacred Heart Hospital and Washington University in St. Louis School of Medicine. The data comprise patients of different ethnic and racial backgrounds. The DDSM contains descriptions of mammographic lesions in terms of the American College of Radiology breast imaging lexicon called the Breast Imaging Reporting and Data System (BI-RADS). Mammograms in the DDSM database were digitized by different scanners depending on the institutional source of the data and have resolutions between 42 and 50 microns. A subset of DDSM cases was selected for this study. Cases with mass lesions were chosen by selecting reports that only included the BI-RADS descriptors for mass margin and mass shape [17].

Through the coordinates provided by the database, a ROI was selected for each image containing the tissue. In some mammogramms we found more than one mass; in these cases, we extracted more than one ROI. For the normal mammograms were selected ROIs of different sizes and texture randomly. Only the pectoral muscle was not considered as a possible ROI, although tissue and fatty tissue were. All non-mass regions were extracted from cases that did not have a mass region. After that, we applied a histogram equalization, and resized all ROIs to 32 × 32 pixels. For clarity of notation, we represent images as vectors created by concatenating rows of pixels called **x**.

We selected 5090 regions of interest out of 2620 cases, 3240 of which had a mass and 1850 were normal controls. For a better validation of the samples we used the technique of 10-fold cross validation, i.e., data were randomly divided into 10 subsets, each subset had 324 mass samples and 185 non-mass samples. Then we used 509 samples for testing and 4581 for training. From a set of ten groups created, we selected a group for the test and nine for training, repeating the process until we had used all groups as testing set, always using all other groups for training.

### Principal Components Analysis

Principal components analysis (PCA) [18,19] has long been used in efficient coding of various biological signals, like speech [20], ECG [21] and EEG [22]. PCA is a well-known optimal linear scheme for dimension reduction in data analysis. The central idea of PCA is to reduce the dimensionality of a data set while retaining as much as possible the variance of the data set.

Thus in many applications PCA is used as a pre-processing of data, serving as input for other numerical models. The advantage in this case is to reduce the number of parameters of the model immediately following the PCA, improving performance and saving processing time.

To obtain this new representation of data in a smaller size, we must perform the following steps: Subtract the average of the data and calculate the covariance matrix as equation 1(1)

where **x **is the data and *μ *is the mean of the data. The notation ∑ for the covariance matrix is widely used and seems natural because ∑ is the uppercase version of *σ*. It should not be confused with the same symbol used for summation of a series. Then we calculate the eigenvalues and eigenvectors of the matrix ∑ and sort the eigenvectors in descending order according to the eigenvalues. We chose the first eigenvectors because they have higher variance to form the feature vector. Next we derived the new data set using the following formula(2)

where **V **is the matrix with eigenvectors. An assumption made for feature extraction and dimensionality reduction by PCA is that most information of the observation vectors is contained in the subspace spanned by the first *m *principal axes, where *m *<*p *for a *p*-dimensional data space. Therefore, each original data vector can be represented by its principal component vector with dimensionality *m*.

**Figure 2:**
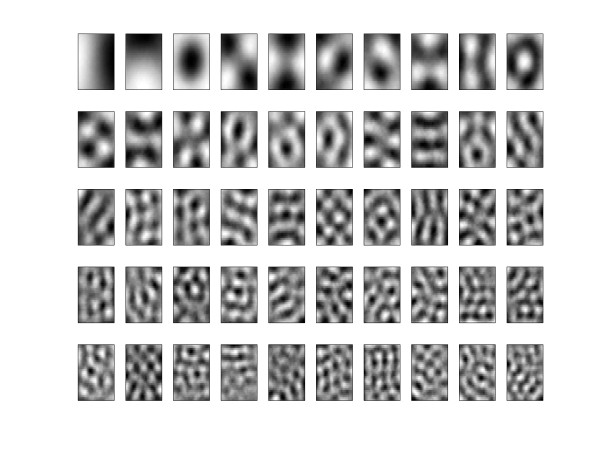
**PCA basis**. The 50 first principal components of ROIs sorted by variance.

Specifically in this work we entered the training set in PCA algorithm and we got as output parameters the principal components, show in Figure 2. Then our training and tests images were projected in these principal components, as in equation 3.(3)

Where in each column of **X***^T ^*is a training image of 1024 × 1 pixels and **V **is a orthogonal matriz 1024 × *k*, which columns represents a principal components and *k *is the number of the selected principal components.

### Efficient Coding

Feature extraction that uses statistics has been heavily influenced by neural information processing models [23]. Neuroscience studies suggested that neuron populations process stimuli information according to the concept of efficient coding [24]. Under this concept, neuron responses are mutually statistically independent which means that there is no redundant information processed throughout the population. The computational aim of efficient coding is to estimate from the statistics of pattern ensemble a compact code that tries to reduce the redundancy in the patterns with minimal loss of information. The data is transformed by a set of linear filters **W **which acts to *X*, and generates **s**. In matrix form(4)

or equivalently in terms of a basis matrix, **x **= **W**^-1^**s **= **As**, where **s **is an estimation of independent components. Methods for deriving efficient code in the model of the equation 4 falls under the rubric of either sparse coding or independent component analysis (ICA) [25].

#### Independent Component Analysis

Let us assume that an image **x **is composed by a set of filters or basis images **A **= [**a_1_**, ⋯, **a_n_**] which are independently activated by coefficients **s **= [*s*_1_, ⋯, *s_n_*]*^T ^*, such as(5)

In 5, only the variables **x **are known, and from them we estimate the coefficients **a **and the mutually independent components **s**.

To estimate several independent components, we used the FastICA algorithm [26], using several units. By a "unit" we refer to a computational unit, eventually an artificial neuron, having a weight vector **w_i _**that the neuron is able to update by a learning rule. The FastICA learning rule finds a direction, i.e. a unit vector **w_i _**such that the projection **w_i_***^T ^***x **maximizes nongaussianity. Nongaussianity is here measured by the approximation of negentropy J (**w***^T ^***x) **given by 6.(6)

The variance of **w_i_***^T ^***x **must be here constrained to unity; for whitened data this is equivalent to constraining the norm of **w_i _**to be unity [27].

**Figure 3:**
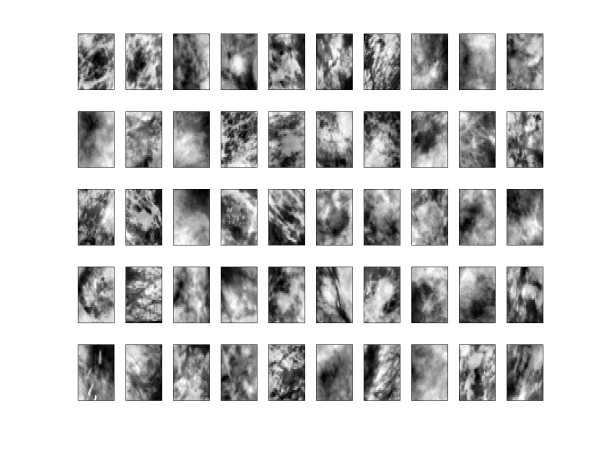
**ICA basis**. The 50 filters generated by the model of efficient coding.

The FastICA was then performed in training set and we obtained 1024 basis functions. To select the most relevant of this basis function we use a similar technique to pursuit, described in the paper by Sousa et. al. [28]. This process consisted of the following steps:

**Step 1: **Define an empty subspace Ψ;

**Step 2: **Repeat next step for *k *= 1, 2, ⋯, *n*, where *n *is the dimension of Ψ;

**Step 3: **Using Eq. 12, classify image  projecting into the subspace composed of [Ψ; *A_k_*], where *A_k _*is the *k^th ^*base function of *A*;

**Step 4: **Select the base functions according to the better result from classification on the training set;

**Step 5: **Move *A_k _*from A to Ψ so that *n *= *n *- 1;

**Step 6: **Return to step 2 until Ψ get the desired dimension;

The 50 most relevant basis function are show in Figure 3.

Then, what we do in the ICA will not be different from what we do in PCA. The set of training and test images are projected in these selected functions basis, as we can see in equation 7.(7)

### Gabor Wavelet

The Gabor filters are band-pass filters with tuneable orientation and radial frequency bandwidths. The Fourier transform of the Gabor filters are Gaussian shifted in frequency. The Gabor representation is proved to be optimal in the sense of minimizing the joint 2-D uncertainty in space and frequency. The Gabor filter kernels have similar shapes as the receptive fields of simple cells in the primary visual cortex when stimulate by naturals image [11]. They are multi-scale and multiorientation kernels. These filters can be represented as the equation 8

A 2-D Gabor function is described as follows:(8)

**Figure 4:**
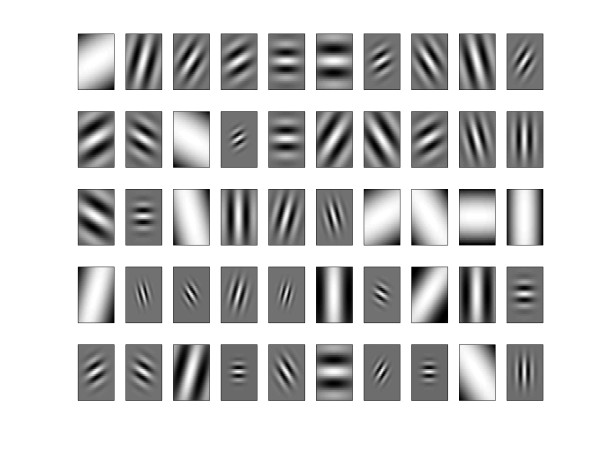
**Gabor basis**. The 50 Gabor wavelet filters chosen by selection algorithm.

A bank of Gabor filters can be obtained by scale and rotation of g(x, y).(9)

where *θ *= *nπ */*k *and *k *is the total number of orientations, *m *= 1, ..., *M*, *M *is the number of scales. With Gabor, we create 100 filters, ten diferents scales and orientations. With the same algorithm used above we perform the selection of Gabor wavelets of which have greater significance in the classification of ROIs. The 50 most significant may be seen in the Figure 4. Then, we projected the set of training and test in these selected gabor filters, as we can see in equation 10.(10)

### Linear Discriminant Analysis

Linear discrimination, as the name suggests, looks for linear combinations of the input variables that can provide an adequate separation for a given class. Rather than looking for a particular parametric form of distribution, LDA uses an empirical approach to define linear decision plans in the attribute space i.e. it models a surface. The discriminant functions used by LDA are built up as a linear combination of the variables that seek to maximize the differences between the classes [29]:(11)

The problem then is reduce to find a suitable vector *β*. There are several popular variations of this idea, one of the most successful being the Fisher Linear Discriminant Rule.

Fisher's Rule is considered a "sensible" classification, in the sense that it is intuitively appealing. It makes use of the fact that distributions that have a greater variance between their classes than within each class should be easier to separate. Therefore, it searches for a linear function in the attribute space that maximizes the ratio of the between-group sum-of-squares **B **to the within-group sum-of-squares **W_LDA_**. This can be achieved by maximizing the ratio(12)

and it turns out that the vector that maximizes this ratio,*β*, is the eigenvector corresponding to the largest eigenvalue of **W_LDA_B **i.e. the linear discriminant function *y *is equivalent to the first canonical variate. Hence the discriminant rule can be written as:(13)

where  and , and *n_i _*is class *i *sample size, ∑*_i _*is class *i *covariance matrix, *x_i _*is the class *i *mean sample value and *x *is the population mean. We use this technique to classify the news test sets . Then for each test set was used corresponding training set, ie, was used to  with ,  with  and was used  with . Each training and testing group is composed of masses (benign and malignant) and non-masses samples.

We chose the LDA for simplicity of implementation and low computational consumption compared to other classifiers such as support vector machine (SVM). We can see in previous works [30] that the SVM has a rate of accuracy greater than the LDA, but the time taken to determine the best parameters in training is higher than the LDA.

### Validation of the Classification Methods

In order to evaluate the classifier with respect to its differentiation ability, we have analyzed its sensitivity, specificity and accuracy. Sensitivity indicates how good the test is to identify the disease and is defined by *TP */(*TP *+ *FN*), specificity indicates how good the test is to identify patients without pathologies and is defined by *TN*/(*TN *+ *FP *), and accuracy is defined by (*TP *+ *TN*)/(*TP *+ *TN *+ *FP *+ *FN*), where *TP *is true-positive, *TN *is true-negative, *FN *is false-negative, and *FP *is false-positive. True-positive means mass samples correctly classified as mass. The meaning of the other ones is analogous.

To determine accuracy, sensitivity and specificity, we used the average of results obtained by 10-fold cross validation.

## Results

**Figure 5:**
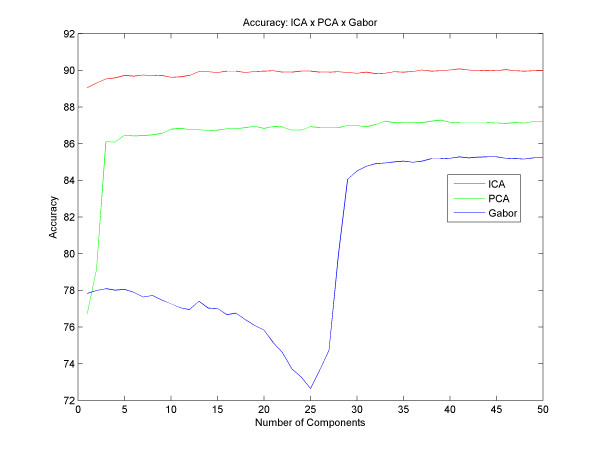
**Accuracy**. Results with different numbers of components and the difference between the accuracy of PCA, ICA and Gabor.

**Figure 6:**
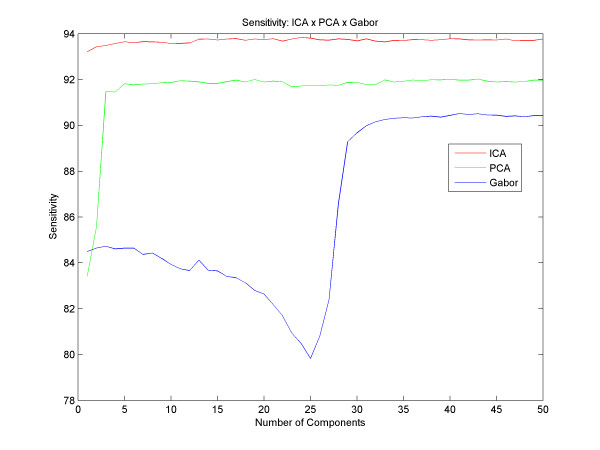
**Sensitivity**. Results with different numbers of components and the difference between the sensitivity of PCA, ICA and Gabor.

**Figure 7:**
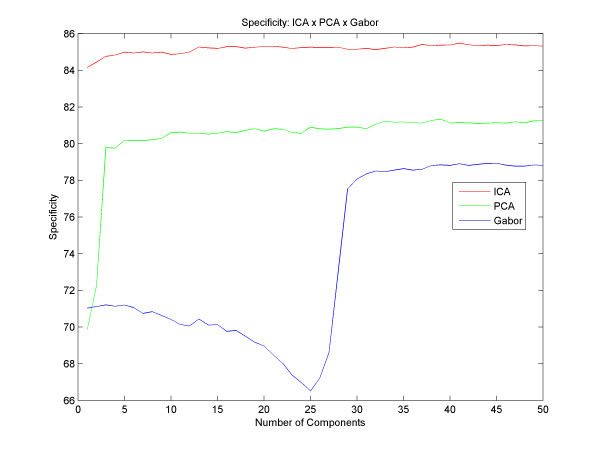
**Specificity**. Results with different numbers of components and the difference between the specificity of PCA,ICA and Gabor.

The best result with PCA obtained an accuracy of up to 87.28% with 39 principal components and with Gabor we got an accuracy of up to 85.28% with 41 components. With the ICA, we obtained an average success rate of 90.07% with 41 independent components. Figure 5 shows different results for different quantities of components, for all techniques described here. However, we could analyze and compare various accuracy results between the different techniques, and observed that the ICA reaches better results than the PCA from the very first component, and that the results with the PCA get better as the number of components increases until the moment of convergence, around the 5th component. Gabor has a different increasing rate for the PCA and the ICA: it starts with a decay in its accuracy until 25 filters and then the success rate increases until it reaches its peak at 41 components and remains stable thereafter. Regarding the sensitivity and specificity, Figure 6 and 7 respectively, we found that using the technique of ICA we obtained better results than using PCA and Gabor (93.83% sensitivity with 24 basis functions and 85.48% specificity with 41 basis). With this result we observe that the system ranks true positive cases better than true negative cases, ensuring good reliability for clinical cases.

**Figure 8:**
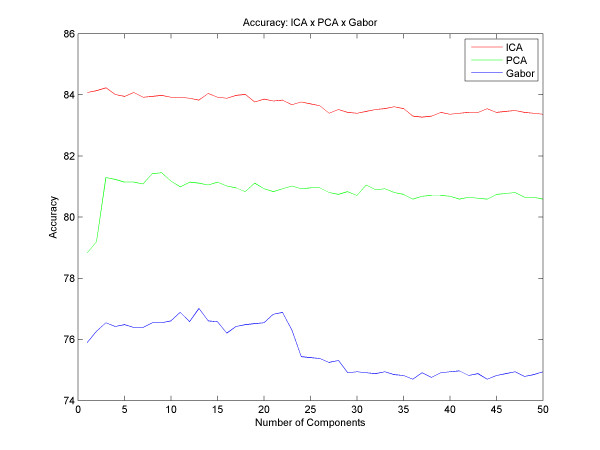
**Accuracy - Benign or Malign**. Results of classification as benign or malign with different numbers of components and the difference between the accuracy of PCA, ICA and Gabor.

**Figure 9:**
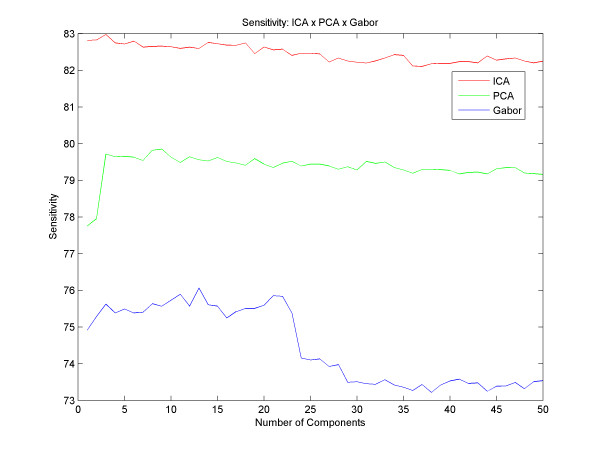
**Sensitivity - Benign or Malign**. Results of classification as benign or malign with different numbers of components and the difference between the sensitivity of PCA, ICA and Gabor.

**Figure 10:**
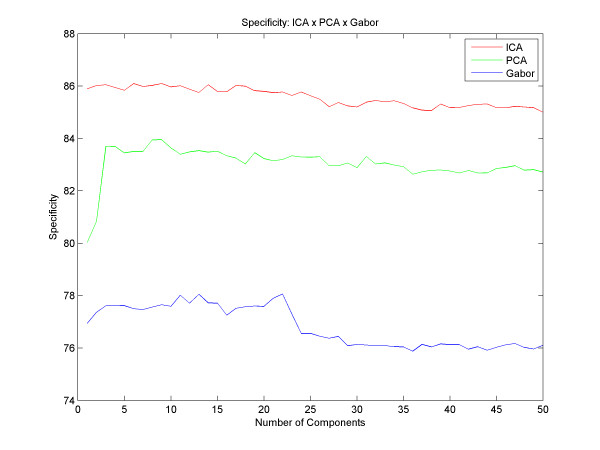
**Specificity - Benign or Malign**. Results of classification as benign or malign with different numbers of components and the difference between the specificity of PCA, ICA and Gabor.

Another study that was conducted is related to the diagnoses of nodules as benign or malignant. We used the same filter banks to obtain the classification of mass and non-mass, and the same techniques for the classification of these nodules. We used 1540 benign mass images and 1700 malign mass images. The results we obtained showed an average accuracy of 84.22% with three components using ICA filters. The sensitivity was 82.97% with three components and specificity was 86.09% with 9 components. PCA resulted in an average accuracy of 81.45%, 79.85% of sensitivity and 83.95% of specificity, all with nine components. Gabor resulted in a success rate of 77.00% and 76.06% of sensitivity both with 13 components and 78.06% of specificity with 22 components. These results can be observed in Figures 8, 9 and 10.

## Discussion and Conclusions

This paper has presented a computer aided diagnosis system based on feature extraction and inspired by the concept of efficient coding, applied to the problem of recognizing breast cancer in ROIs, classifying as mass or non-mass, and in the case of mass further classify as benign or malignant. To perform the classifications we used the Linear Discriminant Analysis.

The improvement using efficient coding is by a few percentage points in sucess rate. Although relatively small, the improvemente is likely to be very valuable, because the occurrence of false negatives (low sensitivity) can lead to human death.

LDA succeeded partially in separating the two classes, but there is still a margin of intersection between them, an area that characterizes the misclassification. In [30] we can see that the hyperplane generated by the Support Vector Machine (SVM) separates these classes better thus providing a better result in the classification. However, the computational cost of the LDA is lower than the SVM, saving time in the operation.

Furthermore this assumption of linearity leads to a limitation of our system, which does not allow us to consider nonlinear structures in feature extraction and classification. In future work we will use nonlinear methods of feature extraction, such as Kernel PCA [31], nonlinear Hidden Markov Models [32] and other statistical models [33,34] in order to achieve a possible improvement in success rates.

An interesting factor in these results is the fact that the best accuracy is not ways achieved using all the components. We suspect that this happens when we use too much information to classify, creating redundancies and confusing the classifier, consequently decreasing the rate of success. We believe the ideal number of components is between 30 and 50, because tests conducted with more components did not achieve the best results, although still got results around average.

Altogether, the results presented demonstrate that independent component analysis performed successfully the efficient coding in order to discriminate mass from non-mass tissues. In addition, we have observed that LDA with ICA bases showed high predictive performance for some datasets and thus provide significant support for a more detailed clinical investigation.

## Competing interests

The authors declare that they have no competing interests.

## Authors' contributions

DDC participated in all stages of the article, provided the theoretical basis to the results and conclusions. LFC participated in the study of feature extraction and classification. AKB participated in its design and coordination and helped to draft the manuscript. All authors read and approved the final manuscript.
